# Seroprevalence of Dengue and Chikungunya Virus Infections in Children Living in Sub-Saharan Africa: Systematic Review and Meta-Analysis

**DOI:** 10.3390/children10101662

**Published:** 2023-10-07

**Authors:** Neema Kayange, Duncan K Hau, Kevin Pain, Stephen E Mshana, Robert Peck, Stephan Gehring, Britta Groendahl, Philip Koliopoulos, Baraka Revocatus, Evarist B Msaki, Ombeva Malande

**Affiliations:** 1 Department of Pediatrics, Bugando Medical Centre, Weill Bugando School of Medicine, Catholic University of Health and Allied Sciences, Mwanza P.O. Box 1464, Tanzania; rnp2002@med.cornell.edu; 2 Department of Pediatrics, Weill Cornell Medical College, New York, NY 10065, USA; dkh2001@med.cornell.edu; 3 Samuel J. Wood Library and C.V. Starr Biomedical Information Center, Weill Cornell Medical College, 1300 York Avenue, New York, NY 10065, USA; kjpain@med.cornell.edu; 4 Department of Microbiology and Immunology, Weill Bugando School of Medicine, Catholic University of Health and Allied Sciences, Mwanza P.O. Box 1464, Tanzania; mshana72@bugando.ac.tz; 5 Center for Global Health, Department of Medicine, Weill Cornell Medical College, New York, NY 10065, USA; 6 Department of Pediatrics, University Medical Center of the Johannes Gutenberg University, 55131 Mainz, Germany; stephan.gehring@uni-mainz.de (S.G.); groendahl@uni-mainz.de (B.G.); philip.koliopoulos@unimedizin.mainz.de (P.K.); 7 Department of Data and Statistics, Bugando Medical Centre, Mwanza P.O. Box 1370, Tanzania; revocatus.baraka@bmc.go.tz; 8 Department of Epidemiology and Biostatistics, Bugando Medical Centre, Mwanza P.O. Box 1370, Tanzania; evarist.msaki@bmc.go.tz; 9 East Africa Centre for Vaccines and Immunization (ECAVI), Kampala P.O. Box 3040, Uganda; ombevaom@e-cavi.com; 10 Department of Public Health Phamarmacy, Sefako Makgatho Health Sciences University, Pretoria P.O. Box 60, South Africa; 11 Department of Paediatrics & Child Health, Makerere University, Kampala P.O. Box 7072, Uganda; 12 Department of Public Health, UNICAF University, Lusaka P.O. Box 20842, Zambia

**Keywords:** prevalence, dengue virus, chikungunya virus, Sub-Saharan Africa, systematic review

## Abstract

Dengue and chikungunya viruses are frequent causes of malarial-like febrile illness in children. The rapid increase in virus transmission by mosquitoes is a global health concern. This is the first systematic review and meta-analysis of the childhood prevalence of dengue and chikungunya in Sub-Saharan Africa (SSA). A comprehensive search of the MEDLINE (Ovid), Embase (Ovid), and Cochrane Library (Wiley) databases was conducted on 28 June 2019, and updated on 12 February 2022. The search strategy was designed to retrieve all articles pertaining to arboviruses in SSA children using both controlled vocabulary and keywords. The pooled (weighted) proportion of dengue and chikungunya was estimated using a random effect model. The overall pooled prevalence of dengue and chikungunya in SSA children was estimated to be 16% and 7%, respectively. Prevalence was slightly lower during the period 2010–2020 compared to 2000–2009. The study design varied depending on the healthcare facility reporting the disease outbreak. Importantly, laboratory methods used to detect arbovirus infections differed. The present review documents the prevalence of dengue and chikungunya in pediatric patients throughout SSA. The results provide unprecedented insight into the transmission of dengue and chikungunya viruses among these children and highlight the need for enhanced surveillance and controlled methodology.

## 1. Introduction

Arboviruses, including dengue [DENV) and chikungunya (CHIKV), are an increasingly important health treat; recurring disease outbreaks have attracted global attention [1,2]. DENV and CHIKV are spreading from their original niche in Sub-Saharan Africa (SSA) and Asia to new areas of the world; the World Health Organization (WHO) recently signaled the danger of the rising spread of CHIKV and DENV in the Americas [3,4]. DENV and CHIKV belong to the *Flaviviridae* and *Togaviridae* families, respectively. Both viruses can be transmitted via a mosquito vector (often *Aedes aegypti* or *Aedes albopictus*) [2,5]. 

Dengue caused by one of four DENV serotypes is the most extensively spread mosquito disease; it is a significant health threat that affects approximately 390 million people annually [1,5]. A 30-fold increase in DENV infections in humans was observed over the last 50 years, with more than 100 million symptomatic dengue cases in patients residing in >128 countries [6,7]. Children have a higher risk than adults of developing the complications of the disease, which include hemorrhage and dengue shock [8].

CHIKV was first described in the Makonde area of southern Tanzania following an outbreak of dengue fever in 1952 [9]. Since its first description, CHIKV has infected millions of people globally [10,11]. Cases of chikungunya have been reported in more than 100 countries [12]. Clinical symptoms of chikungunya include high fever, myalgia, polyarthralgia, and maculopapular rash. Because of their chronic nature, some CHIKV infections can lead to neurological signs or fulminant hepatitis [13]. 

A clear understanding of the frequency of arbovirus infections in African children will enable an estimation of the prevalence of DENV and CHIKV and the necessity for vaccine development. Studies show that an approximate 80% of individuals infected by DENV and CHIKV will remain asymptomatic throughout the course of the infection. This results in underestimation and underreporting in most SSA countries when infections are determined by passive surveillance [7,14].

No treatment or cure for either DENV or CHIKV infection exists. There is a vaccine for dengue; several additional vaccines are in development [15,16,17]. Information regarding the prevalence and geographical distribution of dengue and chikungunya is necessary in order to make decisions concerning the appropriate use of existing and emerging strategies for prevention and control. In this regard, an increased number of studies that describe the prevalence of dengue and chikungunya and the frequency of viral outbreaks in Africa were reported over the course of the last 10 years. Despite this increase, the burden and epidemiology of DENV and CHIV infections in children are not well documented. This is due, in part, to similarities in the clinical presentation of dengue, chikungunya, and other febrile illnesses such as malaria in most African countries and the lack of laboratory capacity to differentiate between them. Consequently, a systematic review and meta-analysis of existing publications was performed here to determine the seroprevalence of DENV and CHIKV infections in children living in SSA. 

## 2. Materials and Methods

### 2.1. Literature Search 

A systematic review in this study was performed in accordance with the Preferred Reporting Items for Systematic Reviews and Meta-Analyses (PRISMA) guidelines [18]. Permission to write the protocol for this review was registered with the Catholic University of Health and Allied Sciences ethical committee (Clearance Certificate no. CREC/455/2020).

### 2.2. Inclusion and Exclusion Criteria

In this systematic review, the following inclusion criteria were used: (1) the enrollment of pediatric patients between 1 month and 19 years of age (sub-analysis of children was conducted for studies that included both children and adults); (2) DENV and CHIKV infections evaluated using PCR and RT-PCR or serology (IgG/IgM ELISA, plaque-reduction neutralization test [PRNT], neutralizing antibodies), and antigen-specific tests, i.e., non-structural protein 1 antigen-rapid diagnostic test [NSI-RDT] and indirect fluorescence antibody [IFA]; (3) black African subjects living in SSA; and (4) multicounty surveys and published conference abstracts that reported analyzed data for the pediatric population only. Notably, the prevalence of dengue in the non-black population is well known [17]. As such, only black Africans were included in this analysis. 

Studies were excluded with the following criteria: (1) no sub-analyses were conducted on pediatric patients for studies that enrolled both adults and children; (2) the study enrolled fewer than 10 participants; (3) publication types were review articles, letters to the editor, or commentaries; or (4) research was unrelated to prevalence/incidence, risk/prognostic factors, or clinical outcomes (e.g., vaccines, immunizations, basic science).

### 2.3. Search Strategy

A medical library information specialist performed a comprehensive search of the MEDLINE (Ovid), Embase (Ovid), and Cochrane Library (Wiley) databases on 28 June 2019, and an updated search on 12 February 2022. The search strategy was designed to retrieve all articles related to arboviruses in African children using both controlled vocabulary and keywords. The searches were limited to English-language articles published between 2009 and the updated search dates. The details of the specific database are provided in Supplementary Table S1. The search was performed according to inclusion and exclusion criteria stated in Section 2.2.

### 2.4. Literature Selection and Data Extraction

Two independent investigators (NK and DH) initially screened titles and abstracts in Covidence (Melbourne, Australia) (a web-based platform for systematic review screening) and then in Excel. Conflicting opinions and uncertainties were discussed and resolved by reaching a consensus with a third reviewer (RP).

NK and DH screened the full text; disagreements were resolved by RP. Data extraction was performed using standardized data extraction Excel forms. The following data from each study were recorded: study title, lead author, publication year, year conducted, village/city/area, country, study period and population, sampling method, laboratory assays, age of participants, gender, presented signs and symptoms, prevalence of DENV and CHIKV, incidence of arbovirus infections, comorbidity, and management outcome. 

### 2.5. Quality Assessment

The quality of each study was evaluated using the Joanna Briggs Institute Critical Appraisal checklist for studies reporting prevalence data, which uses a 9-item checklist [19]. An item was scored “0” if it was answered “no” or “unclear” and “1” if it was answered “yes.” Article quality assessment was defined as follows: high-risk quality = 0–3, moderate risk quality = 4–6, and low risk = 7–9. This systematic review and meta-analysis included 47 studies: 39 (83.0%) studies were of low risk quality, and 8 (17.0%) were of moderate quality (Table S2). No study ranked high-risk quality.

### 2.6. Statistical Analysis

Data analysis was performed using STATA version 15 (STATA Corp LLC., College Station, TX, USA). A meta-analysis was conducted to assess the proportion of arbovirus infections in SSA. A random effect model was used to estimate the pooled (weighted) proportion of dengue and chikungunya; heterogeneity was assessed using Cochran’s Q test (I^2^).

## 3. Results

### 3.1. Study Selection and Characteristics

**Figure 1:**
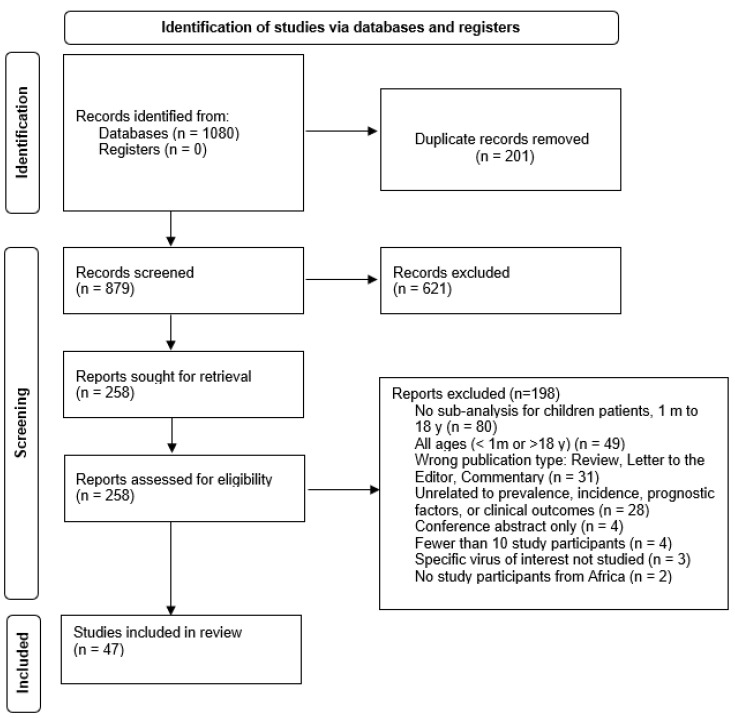
Prisma flow diagram of systematic reviews that included searches of databases and registers only.

The literature search identified 1080 potentially relevant citations; 201 were duplicates leaving, 879 to be screened. A total of 621 articles were excluded at the title/abstract stage, leaving 258 articles for full-text review. After reviewing the full text, 198 articles met the exclusion criteria. Figure 1 shows a PRISMA flowchart of the study selection process [19]. Forty-nine articles were included in this review [20,21,22,23,24,25,26,27,28,29,30,31,32,33,34,35,36,37,38,39,40,41,42,43,44,45,46,47,48,49,50,51,52,53,54,55,56,57,58,59,60,61,62,63,64,65,66,67,68]. 

Forty-seven articles that described studies published between 2009 and 2021 in SSA were eligible for systematic review. The majority of these studies (22) were conducted in East African countries that included Tanzania (n = 7 studies), Kenya (n = 12), and Mozambique (n = 3). Thirteen studies were conducted in the West African countries of Nigeria (n = 6), Burkina Faso (n = 2), Senegal (n = 4), and Ghana (n = 1). A few were conducted in the North African country of Sudan (n = 4) and the Central African countries of Camerron (n = 4), Congo (n = 2), and Gabon (n = 2). This review involved 25,186 participants. Evidence of arbovirus infection was reported for DENV and CHIKV in 39 and 23 datasets, respectively (Table 1). No study originating in South Africa was reported. Urban areas exhibit a higher seroprevalence of dengue and chikungunya, 19.3% (95% CI: 11.2, 28.9) and 11.2% (95% CI: 5.1, 19.2), respectively, than did rural areas, 12.1% (95% CI: 5.27, 21.0) and 4.6% (95% CI: 2.8, 6.8), respectively (Table S3). Studies conducted in hospital settings reported a higher seroprevalence of dengue, 18.4% (95% CI: 11.9, 25.8), compared to community settings, 6.1% (95% CI: 0.0, 25.9). The seroprevalence of chikungunya was the same for studies performed in hospital and community settings (Table S3).

### 3.2. Prevalence of DENV and Geographic Coverage

**Figure 2:**
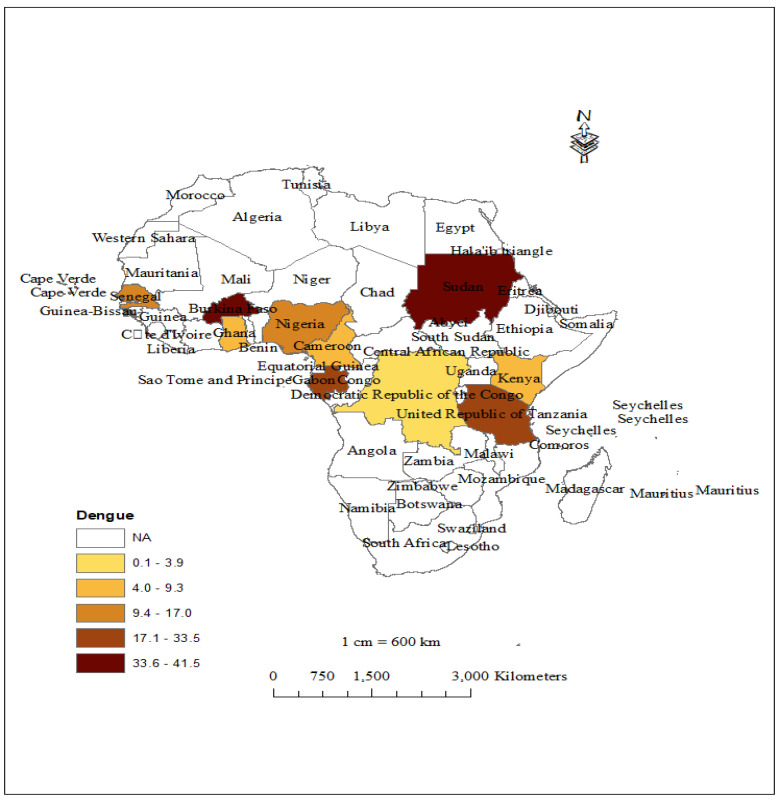
Map showing areas of DENV infection in children living in SSA.

**Figure 3:**
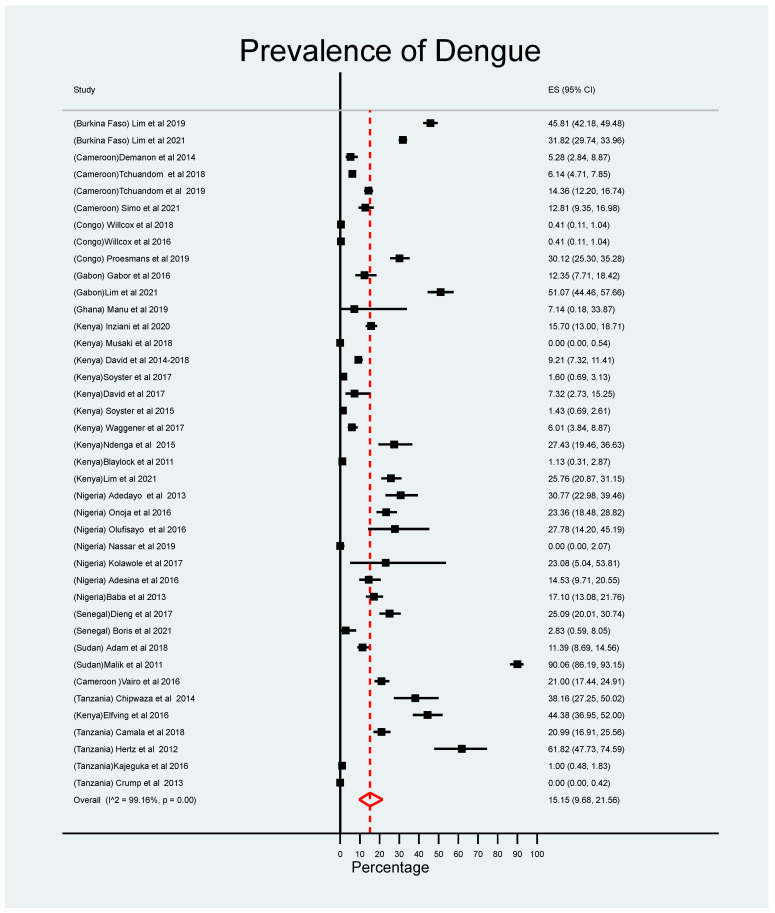
Estimated meta-analysis of the prevalence of DENV in febrile children living in SSA. Midpoint of each horizontal line segments represents the proportional of prevalence of dengue for each study while the rhombic mark shows the pooled proportions for all studies [20,22,23,24,25,27,28,29,30,31,32,33,34,35,36,39,40,41,42,43,44,45,46,47,48,49,50,51,54,55,57,58,59,60,61,63,64,65,66].

**Figure 4:**
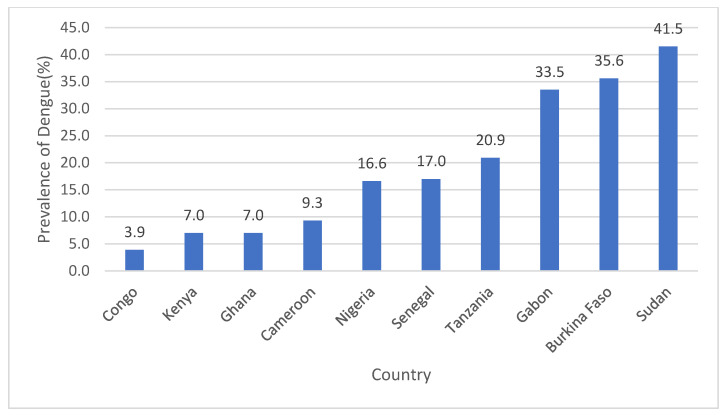
Prevalence of DENV infections in children by SSA country.

The prevalence of DENV infections in children reported in 39 studies was widely distributed in East African countries (i.e., Tanzania and Kenya); a few countries in West Africa, including Cameroon and Burkina Faso, also reported infections (Figure 2). Of the 47 studies included in this review, only 39 provided information about the proportion of laboratory-confirmed dengue cases among patients diagnosed clinically. The overall pooled prevalence of DENV infections among SSA countries was estimated to be 15.79% (95% CI: 10.17, 22.34) (Figure 3). Countries that reported the highest prevalence of infections were Sudan (41.5%), Burkina Faso (35.6%), Gabon (33.5%), and Tanzania (20.9%); the lowest prevalence of dengue was observed in the Congo (3.9%) (Figure 4). Subgroup analysis shows a declining trend in DENV infections over time. The prevalence of DENV infections was lower during the 2010–2020 period than the 2000–2009 period, i.e., 12.66% (95% CI: 7.64, 18.69) versus 30.90% (95% CI: 0.58, 78.66), respectively (Supplementary Figure S1a,b). 

### 3.3. Prevalence of Chikungunya and Geographical Coverage

**Figure 5:**
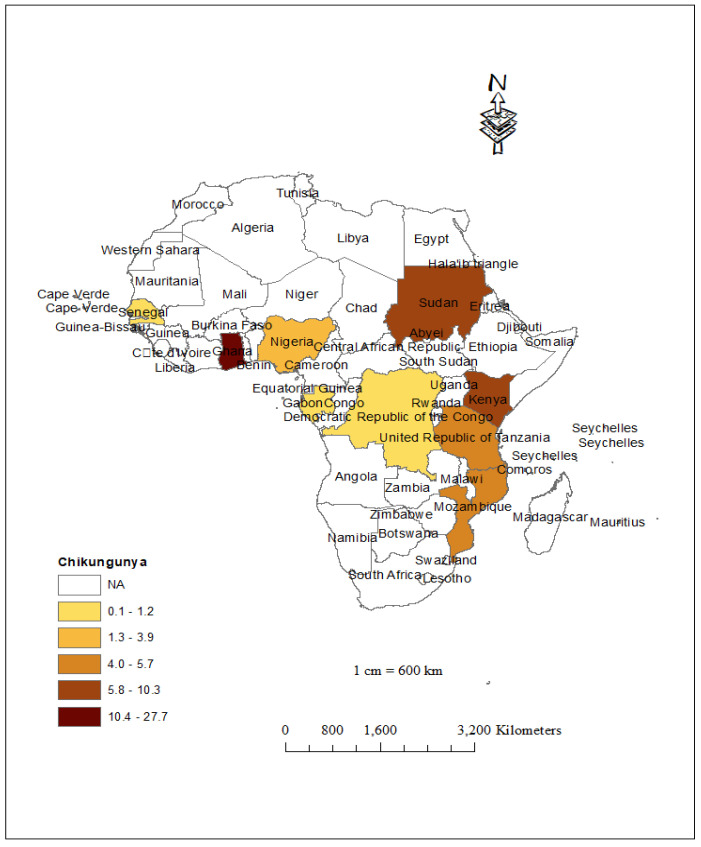
Areas of CHIKV infection in children living in SSA.

**Figure 6:**
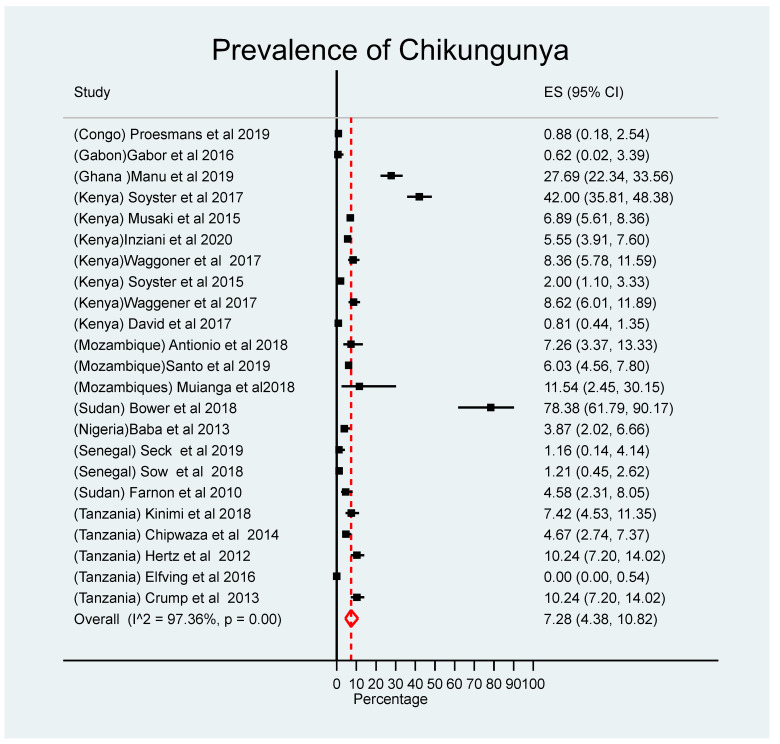
Estimated meta-analysis of the prevalence of CHIKV infections in febrile children living in SSA. Midpoint of each horizontal line segments represents the proportional of prevalence of Chikungunya for each study while the rhombic mark shows the pooled proportions for all studies [20,21,22,26,27,28,32,33,34,35,36,37,38,41,45,49,52,53,56,62,63,66].

**Figure 7:**
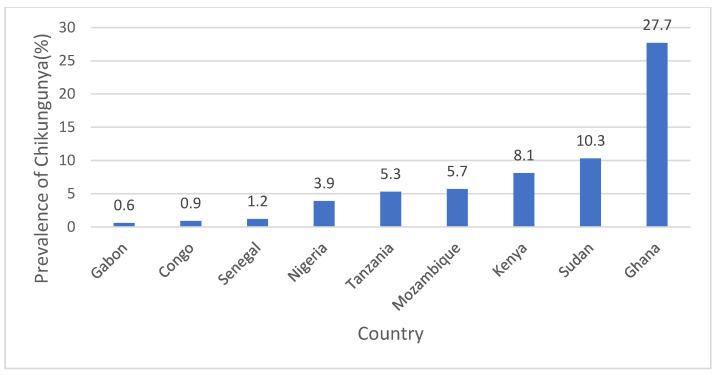
Prevalence of CHIKV infections in children by country in SSA.

CHIKV infections were widely distributed in the East (Tanzania, Kenya, and Mozambique), Central (Congo and Gabon), and West (Senegal and Nigeria) African countries. A few North African countries (i.e., Sudan) reported infections (Figure 5). Of the 47 studies included in this review, 23 provided information about the proportion of laboratory-confirmed chikungunya cases among clinically suspected patients; these were included in the meta-analysis. The overall pooled prevalence of chikungunya in African countries was 7.28% (95% CI: 5.66, 16.59) (Figure 6). Countries that reported the highest prevalence were Ghana (27.7%) and Sudan (10.3%) (Figure 7); countries that reported the lowest were Congo (0.9%) and Gabon (0.42%). The prevalence of CHIKV infections was lower during the 2000–2009 period, 5.35% (95% CI: 2.29, 9.53), than in the 2010–2020 period, 7.96% (95% CI: 4.25, 12.64) (Figure S2a,b). 

### 3.4. Pooled Prevalence of DENV and CHIKV by Region

Table 2 depicts the overall prevalence of dengue, 17.02% (95% CI: 11.79, 22.95), which was reportedly higher in most regions of Africa than chikungunya, 10.51% (95% CI: 5.66, 16.59). Additionally, North and West Africa exhibit a higher prevalence of dengue (30.78% and 24.70%, respectively) compared to other regions. Surprisingly, some regions of East Africa with a low dengue prevalence (10.70%) exhibit a higher pooled chikungunya prevalence (11.97%) compared to other regions.

### 3.5. Laboratory Method Used to Diagnose Arbovirus Infections

Arbovirus infections in the studies reviewed here were diagnosed by the following methods: (1) PCR (RT-PCR, PCR), (2) serology (IgG/IgM-ELISA, plaque-reduction neutralization test [PRNT], neutralizing antibodies), and (3) antigen detection (NSI-RDT, IFA) (Table S4). The gold standard for diagnosing arbovirus infections is PCR, or RT-PCR, used in the majority of studies. Of the 47 reviewed papers, only 39 and 23 reported the prevalence of dengue and chikungunya, respectively. The prevalence in each study was diagnosed by a combination of methods (Table 3). The prevalence of DENV infections diagnosed by PCR, IgG-ELISA, and IgM-ELISA was 12.7% (95% CI: 4.4, 24.5), 6.6% (95% CI: 2.8, 11.6), and 26.6% (95% CI: 16.7, 37.8), respectively, while the prevalence of CHIKV infections was 8.8% (95% CI: 2.6, 18.2), 7.4% (95% CI: 2.8, 13.8), and 8.2% (95% CI: 5.0, 12.2) diagnosed by PCR, IgG-ELISA, and IgM-ELISA, respectively (Figure S3).

### 3.6. DENV and CHIKV Co-Infections

DENV and CHIKV are able to co-infect humans as a consequence of their common routes of transmission by *Aedes* mosquitoes and similar epidemiological patterns. Sixteen studies in this review reported DENV and CHIKV co-infections [21,22,27,28,29,34,36,41,45,63]. Dengue and chikungunya have common, overlapping symptoms, making them difficult to distinguish clinically. In resource-limited settings, patients are often only tested for DENV infections; consequently, CHIKV infections go undiagnosed. As such, use of the appropriate laboratory methods is mandatory for correct diagnoses.

## 4. Discussion

The prevalence of DENV and CHIKV infections in black SSA children was reported in this review of 47 published articles. The results indicate that these infections have spread throughout SSA; the prevalence of DENV and CHIKV was comparatively well documented in 39 and 23 studies, respectively. The pooled prevalence of DENV infections in symptomatic children with fever was ~16%; the highest prevalence was reported in Sudan among children during outbreaks [50] and also in Tanzania and Burkina Faso, mostly diagnosed in outpatient settings [22,60,61]. The pooled prevalence of chikungunya was 7% [21,22,27,53]. DENV and CHIKV infections often occurred in clinical settings that did not include the differential diagnosis of dengue and chikungunya in children presenting with fever; this frequently resulted in unnecessary treatment with antimalarials and antibiotics [22]. Most clinicians in SSA rely on judgment that often depends upon epidemiological data, which is lacking in the majority of settings. There are no well-established epidemiological surveillance systems or laboratory diagnostic approaches. Therefore, reported cases are often sporadic and confused with other febrile illnesses like malaria, typhoid fever, and HIV infections, which are very common in children and endemic in Africa [35,65]. Correct diagnoses require laboratory confirmation.

Systematic review and meta-analysis studies conducted in Africa reported that the pooled prevalence of DENV infection ranged from 10 to 62%, dependent upon the method of diagnosis and period of outbreak or non-outbreak [67,68,69]. These studies, however, failed to differentiate between the prevalence in children and adults. Children are more susceptible to severe diseases such as dengue hemorrhagic fever and dengue shock syndrome [5]. One study conducted in Africa reported that the pooled prevalence of chikungunya determined by IgM- and IgG-ELISA was 9.7% and 16%, respectively [70]. These values are higher than those reported in the current study, which only included children and adolescents. The significant presence of dengue and chikungunya in SSA aligns with the spread of these arboviral diseases worldwide, especially in the Americas [3,4]. The WHO recently reported an alarming rise in dengue and chikungunya cases in the Americas, with a sharp rise every three to five years. The suspected and confirmed cases of arboviral diseases increased to 3,123,752 in 2022: 2,809,818 cases of dengue (90%) and 273,685 cases of chikungunya (9%). This increase represents an approximate 119% increase compared to cases of arbovirus infections in 2021 in endemic parts of South America, Central America, and the Caribbean. This worrisome trend requires further study, especially with respect to the shift from traditional patterns of spread and transmission. The WHO is actively investigating the: high risk of spread, evolution of vector mosquitoes, altered biting patterns, changing clinical presentations and management, increased rates of prolonged disease and death, expansion of diseases beyond historic areas of transmission, and the spread of disease in immunologically-naïve populations. Related conclusions were gathered from the large systematic review and meta-analysis reported here, looking at arbovirus infections and the occurrence of DENV and CHIKV coinfections. 

A variety of serological methods reported here are used to test for DENV and CHIKV infections; ELISA IgG/IgM is the most common. Virus-specific IgM can be detected 4–5 days after the onset of illness since viremia and antigenemia typically occur between 2 and 3 days prior to the onset of fever and last for 2–7 days of illness [71]. ELISA IgG, however, is generally recommended due to its increased sensitivity, stronger response, and persistence of antigen-specific IgG in sera [72]. It is less specific, though, due to the cross-reactivity between arboviruses that occurs due to similarities in the amino acid sequence of homologous proteins found in different flaviviruses, especially DENV and ZIKV [71,73]. 

The dual prevalence of DENV and CHIKV reported in Mozambique, Tanzania, and Kenya in our study suggests overlapping endemicity [22,23,39]. This occurrence was examined in the extensive global systematic review and meta-analysis that found a pooled global coinfection prevalence of 2.5% for DENV and CHIKV coinfection [3,4]. Acute infections can be confirmed by detecting viral genomes or by antigen tests. Other serological methods, such as immunofluorescence, rapid diagnostic, and virus neutralization tests, also detect anti-DENV antibodies. Rapid antibody tests, available though not used routinely in most African countries, can even be used by laboratories with limited technical facilities during outbreaks. Limited resources in many SSA countries prohibit the diagnosis of many arbovirus infections using RT-PCR or other genome detection systems [74]. The extent to which differences in the methods of testing, as well as coinfections with DENV and CHIKV, contribute to the varying prevalence of dengue and chikungunya in the same country, or region requires further examination and evaluation. Also, these heterogeneous results across regions may also be related to climatologic factors and population density (urban vs. rural), but likely differences are due to limited facilities and inadequate testing capacity.

Currently, there are no curative therapies, i.e., anti-viral drugs, available to treat either DENV or CHIKV infections in children [5,75,76]. The majority of cases are managed with rest and aggressive supportive treatment; hospitalization is needed only in severe cases. Therefore, correct diagnoses that make supportive care quickly available, exclude treatable diseases, and identify regional clusters of infections are imperative. Notably, fifty percent of dengue and chikungunya cases are incorrectly treated with antibacterial and antimalarial drugs because few clinics have the expertise and financial means required to test for arboviral infections [22,35,59]. Rather, clinical assessment often relies upon epidemiological information regarding the likelihood of the presence of a specific disease in the area. Continuous monitoring will allow the detection of emerging arbovirus outbreaks and enable the immediate notification and preparation of healthcare workers for incoming cases. Such large-scale outbreaks are documented by a history of DENV and CHIKV infections that occurred over the past two decades [22,24,50,56]. 

In the current review, most children infected with DENV or CHIKV exhibited mild or moderate symptoms and were treated as outpatients [22,23,68]. Severe complications, however, can occur [45,50,56]. Complications, i.e., hemorrhagic fever, shock syndrome, and death, were reported for outbreaks of dengue in children [50]. Reportedly, the prevalence of malaria is declining in SSA, and viral infections are a more frequent cause of febrile diseases in children [77,78]. Apart from any role in respiratory and gastrointestinal tract infections, common arboviruses like DENV and CHIKV need to be considered by clinicians as the potential cause of acute febrile diseases in children living in parts of SSA [22,48,51,62,79,80,81,82]. Awareness and the availability of the appropriate diagnostic tools allow clinicians to diagnose DENV and CHIKV infections early, implement the correct treatment, monitor warning signs for the most severe forms, and establish effective preventive measures.

The frequent occurrence of co-existing dengue and chikungunya cases is reported here in accordance with other reviews [3,4]. As such, the risk of misdiagnosing dengue as chikungunya or failing to diagnose DENV infections altogether is high, thus potentially delaying the treatment of both diseases [82,83,84]. The fact that malaria is endemic in these countries also complicates the diagnosis of dengue and chikungunya since most febrile illnesses in children are assumed to be malaria [85]. This presents a major challenge, especially in terms of vector control strategies. While insecticide-treated bed nets and indoor insecticide spraying are effective in limiting the spread of malaria by *Anopheles* mosquitoes, for example, these measures are far less effective in limiting the transmission of DENV and CHIKV by *Aedes* mosquitoes, which typically bite during the day [86].

The main findings in this review are based upon the widespread seropositivity of DENV and CHIKV infections in children. Questions regarding viral burden, misdiagnosis, and the availability of diagnostic tests need to be addressed. Proper surveillance systems, laboratory testing facilities, clinician diagnostic training to recognize dengue and chikungunya, and vector control strategies are urgently needed. 

This systematic review has several limitations. The study includes data derived from cross-sectional seroprevalence studies and clinic-based surveillances. Only peer-reviewed literature from selected databases was included, thus excluding literature that could have provided additional data. Secondly, most dengue and chikungunya studies were hospital-based; few community-based studies were included. Hospital-based studies do not provide any information concerning disease transmission at the community level since hospitalization is a function of the health-seeking behavior of study participants. The prevalence of dengue and chikungunya was estimated using the number of patients tested in hospitals as the denominator. In some cases, though, it was difficult to determine whether the study involved adults as well as children. Thirdly, most hospital-based studies included in this review used varying case definitions and laboratory tests to confirm DENV and CHIKV infections. Finally, information pertaining to the age and gender of the study participants was not uniformly reported in all studies. Consequently, the prevalence of either dengue or chikungunya was not estimated in terms of these variables.

## 5. Conclusions

DENV and CHIKV infections spread and co-circulate among pediatric patients in some geographical regions of SSA. Children are especially vulnerable to these infections due to the frequency with which they develop the most severe forms [5]. Results from this systematic review and meta-analysis advocate the need for African governments and policymakers to establish routine laboratory measures to diagnose dengue and chikungunya. These should result in guidelines that facilitate earlier diagnosis, implementation of the correct treatment, monitorization of the warning signs of the most severe cases, and the establishment of effective preventive measures. Gaps in the understanding of DENV and CHIKV infections in children living in SSA emphasize the need to initiate community-based studies of child cohorts that represent different geographic regions. These studies will generate reliable estimates of age-specific incidences of dengue and chikungunya and help generate dengue and chikungunya seroprevalence data for children across all SSA.

## Figures and Tables

**Table 1:** Baseline characteristics of 49 arbovirus studies conducted in children in SSA.

First Author	Region	Country	Sample Size (Children)	Sample Size (Adults + Children)	Female n (%)	Range Age <1–19 Years.	Epidemio-Logic Context	DQA Status	DENV	CHIKV	Ref
K. Elfving	Eastern	Tanzania	83	677	NA	0–5	Non-outbreak	H	0(0.0)	0	[20]
A. Muianga	Eastern	Mozambique	26	146	NA	0–19	Outbreak	H	15 (57.7)	5 (19.2)	[21]
A. Chipwaza	Eastern	Tanzania	364	364	178 (48.9)	5–19	Non-outbreak	H	76 (20.9)	17 (4.7)	[22]
B.A. Ndenga	Eastern	Kenya	113	113	NA	2–17	Non-outbreak	M	31 (27.4)	NA	[23]
N. Camara	Eastern	Tanzania	85	362	NA	15–19	Outbreak	H	9 (10.6)	NA	[24]
D.M. Vu	Eastern	Kenya	48	1,863	850 (47.2)	2–17	Non-outbreak	H	11 (22.9)	NA	[25]
E. Kinimi	Eastern	Tanzania	256	728	NA	1–19	Non-outbreak	H	NA	19 (7.4)	[26]
E.N. Grossi-Soyster	Eastern	Kenya	250	500	NA	15–19	Non-outbreak	M	2 (0.8)	141 (56.4)	[27]
E.N. Grossi-Soyster	Eastern	Kenya	968	968	51	1–17	Non-outbreak	H	12 (1.2)	5 (0.5)	[28]
F. Vairo	Eastern	Tanzania	129	481	NA	15–19	Outbreak	M	12 (9.3)	NA	[29]
J.K. Lim	Eastern	Kenya	140	482	NA	1–19	Non-outbreak	H	76 (54.3)	NA	[30]
J.M. Blaylock	Eastern	Kenya	354	354	NA	1–4	Non-outbreak	H	4 (1.1)	NA	[31]
J. Waggener	Eastern	Kenya	383	383	193 (50.4)	0–17	Non-outbreak	H	NA	32 (8.4)	[32]
J.A. Crump	Eastern	Tanzania	332	870	NA	0.2–13	Non-outbreak	H	0	34 (10.2)	[33]
J.T. Hertz	Eastern	Tanzania	712	1447	NA	0–19	Non-outbreak	H	35 (9.2)	34 (10.2)	[34]
M. Inziani	Eastern	Kenya	656	656	340 (51.8)	1–12	Non-outbreak	H	103 (15.7)	36 (5.5)	[35]
S.K. Musak	Eastern	Kenya	1378	1378	NA	2–17	Non-outbreak	M	10 (0.73)	95 (6.9)	[36]
V.S. Antonio	Eastern	Mozambique	124	392	NA	0–19	Non-outbreak	H	NA	12 (9.7)	[37]
V.S. Antonio	Eastern	Mozambique	857	895	NA	0–19	Non-outbreak	H	NA	49 (5.7)	[38]
D.M. Vu	Eastern	Kenya	1104	1104	485 (43.9)	1–17	Non-outbreak	M	82 (7.4)	NA	[39]
D.M. Vu	Eastern	Kenya	1249	1249	NA	1–17	Non-outbreak	M	79 (6.3)	NA	[40]
D.M. Vu	Eastern	Kenya	3835	3835	NA	1–17	Non-outbreak	M	83 (6.2)	14 (0.8)	[41]
A.C. Willcox	Central	Congo	978	978	NR	0.5–5	Non-outbreak	H	4 (0.4)	NA	[42]
H.S. TchetgnaI	Central	Cameroon	75	320	NR	3–19	Non-outbreak	H	8 (10.7)	NA	[43]
J.K. Lim	Central	Gabon	624	682	NR	1–19	Non-outbreak	H	107 (17.1)	NA	[44]
J.J. Gabor	Central	Gabon	162	162	83 (51.0)	3–19	Outbreak	H	31 (19.1)	1 (0.6)	[45]
M. Demanou	Central	Cameroon	435	2030	NA	2–19	Non-outbreak	H	102 (23.4)	NA	[46]
S.B. Tchuandom	Central	Cameroon	961	961	466 (48.5)	0.3–15	Non-outbreak	H	138 (14.4)	NA	[47]
S.B. Tchuandom	Central	Cameroon	961	961	467 (48.6)	0.3–15	Non-outbreak	H	59 (6.1)	NA	[48]
S. Proesmans	Central	Congo	180	342	NA	0–19	Non-outbreak	H	9 (5)	0	[49]
A. Malik	Northern	Sudan	312	312	126 (40.4)	2–15	Outbreak	M	36(90)	NA	[50]
A. Adam	Northern	Sudan	55	483	NA	0–19	Non-outbreak	H	29 (52.7)	NA	[51]
E.C. Farnon	Northern	Sudan	240	552	NA	0–19	Non-outbreak	M	NA	13 (5.4)	[52]
H. Bower	Northern	Sudan	37	155	NA	3–19	Non-outbreak	H	NA	34 (91.9)	[53]
F. Adedayo	Western	Nigeria	130	130	62 (47.7)	2–18	Non-outbreak	H	40 (30.8)	NA	[54]
A.B. Onoja	Western	Nigeria	95	274	NA	0–10	Non-outbreak	H	20 (21.1)	NA	[55]
A. Sow	Western	Senegal	659	1409	NA	0–14	Outbreak	H	NA	6 (0.09)	[56]
O.A. Adesina	Western	Nigeria	15	179	NA	2–19	Non-outbreak	H	5 (33.3)	NA	[57]
H.G. Boris	Western	Senegal	106	106	64 (60.0)	0.3–10	Non-outbreak	H	3 (2.8)	0	[58]
I. Dieng	Western	Senegal	181	960	NA	0–19	Non-outbreak	H	7 (3.9)	NA	[59]
J.K. Lim	Western	Burkina Faso	994	2929	NA	1–19	Non-outbreak	H	210 (21.1)	NA	[60]
J.K. Lim	Western	Burkina Faso	1381	2897	NA	1–19	Non-outbreak	H	611 (44.2)	NA	[61]
M.C. Seck	Western	Senegal	472	1465	NA	0–15	Non-outbreak	H	NA	2 (0.04)	[62]
M. Baba	Western	Nigeria	49	310	29 (59.2)	0–19	Non-outbreak	H	31 (63.2)	24 (49.0)	[63]
S.A. Nassar	Western	Nigeria	42	170	NA	0–19	Non-outbreak	H	0	NA	[64]
O.M. Kolawole	Western	Nigeria	13	176	NA	0–19	Non-outbreak	H	3 (23.1)	NA	[65]
S.K. Manu	Western	Ghana	14	260	NA	6–19	Non-outbreak	H	1 (7.1)	NA	[66]

Key: NA, not available; H, high; M, moderate; DENV, dengue virus; CHIKV, chikungunya virus.

**Table 2:** Pooled prevalence of dengue and chikungunya by region.

Region	Pooled Prevalence (95% CI)	
	Dengue	Chikungunya
Eastern	10.70 (4.70, 18.71)	11.97 (5.45, 20.51)
Western	30.78 (19.23, 43.61)	4.99 (0.07, 15.33)
Central	16.56 (7.49, 28.13)	3.36 (0.25, 8.70)
Northern	24.70 (9.31, 44.36)	10.85 (7.30, 14.94)
Africa	17.02% (95% CI: 11.79, 22.95)	10.51% (95% CI: 5.66, 16.59)

**Table 3:** Diagnostic methods used to determine the prevalence of dengue and chikungunya in children living in SSA.

Diagnostic Method	Dengue, n (%)	Chikungunya, n (%)
IgM-ELISA, IgG-ELISA	29 (74.4)	17 (73.9)
PCR	22 (56.4)	11 (47.8)
RT-PCR	15 (38.5)	8 (34.8)
PRNT	2 (5.0)	1 (4.3)

Key: PCR, polymerase chain reaction; RT-PCR, reverse transcription-polymerase chain reaction; IgG-ELISA and IgM-ELISA, enzyme-linked immunosorbent assays that detect antigen-specific IgG and IgM class antibodies, respectively; NSI-RDT, non-structural protein 1 antigen-rapid diagnostic test; PRNT, plaque-reduction neutralization test.

## Data Availability

All extracted data are included in this paper.

## Supplementary Materials

The following supporting information can be downloaded at https://www.mdpi.com/article/10.3390/children10101662/s1: Table S1. Arboviruses in Sub-Saharan African children combined data search strategies; Table S2. Quality assessment checklist; Table S3. Seroprevalence of dengue and chikungunya by study area (urban vs. rural) and by study site (hospital vs. community); Table S4. Laboratory methods used to diagnose arboviral infections. Figure S1a,b. Funnel plots for the pooled prevalence of DENV infections by periods 2000–2009 and 2010–2020; Figure S2a,b. Funnel plots for the pooled prevalence of CHIKV infections by periods 2000–2009 and 2010–2020. Figure S3. Prevalence of DENV and CHIKV infections by different diagnostic methods used in this study.
