# Supplementary material for: Seroprevalence of Dengue and Chikungunya Virus Infections in Children Living in Sub-Saharan Africa: Systematic Review and Meta-Analysis
Source: Children (Basel). 2023 Oct 7;10(10):1662. doi: 10.3390/children10101662 (PMC10605353; doi:10.3390/children10101662)
Supplement: Supplementary file 1 [file children-10-01662-s001.zip › Figure S1a and b.Funnel plot pooled prevalence of dengue infections by period 2000-2009 and 2010-2020.pdf]

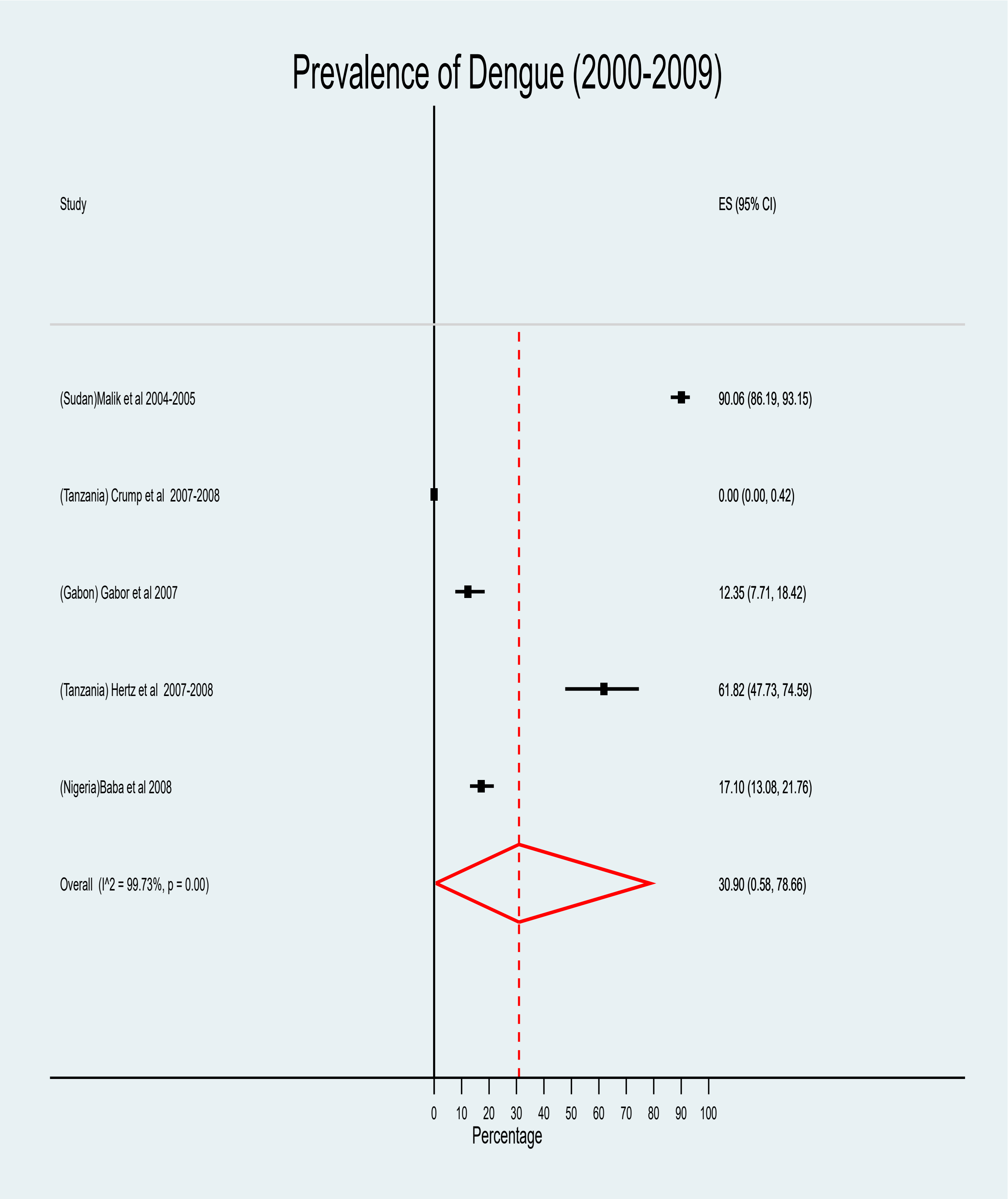

Figure S1a. Forest plot showing the pooled prevalence of dengue from 2000 to 2009.

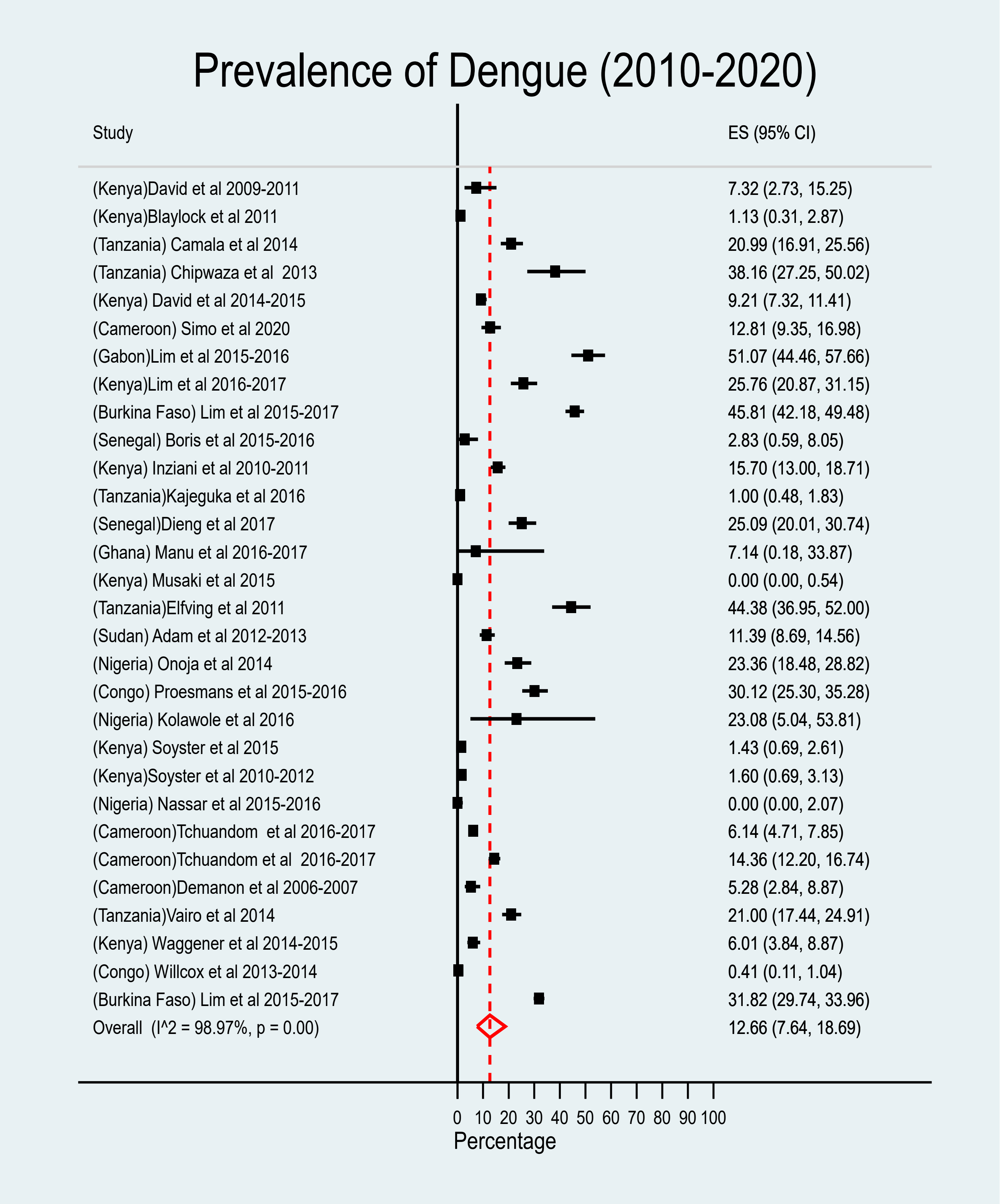

Figure S1b. Forest plot showing the pooled prevalence of dengue from 2010 to 2020.
