# Supplementary material for: Seroprevalence of Dengue and Chikungunya Virus Infections in Children Living in Sub-Saharan Africa: Systematic Review and Meta-Analysis
Source: Children (Basel). 2023 Oct 7;10(10):1662. doi: 10.3390/children10101662 (PMC10605353; doi:10.3390/children10101662)
Supplement: Supplementary file 1 [file children-10-01662-s001.zip › Figure S2a and b. Funnel plot pooled chikungunya infections by period.pdf]

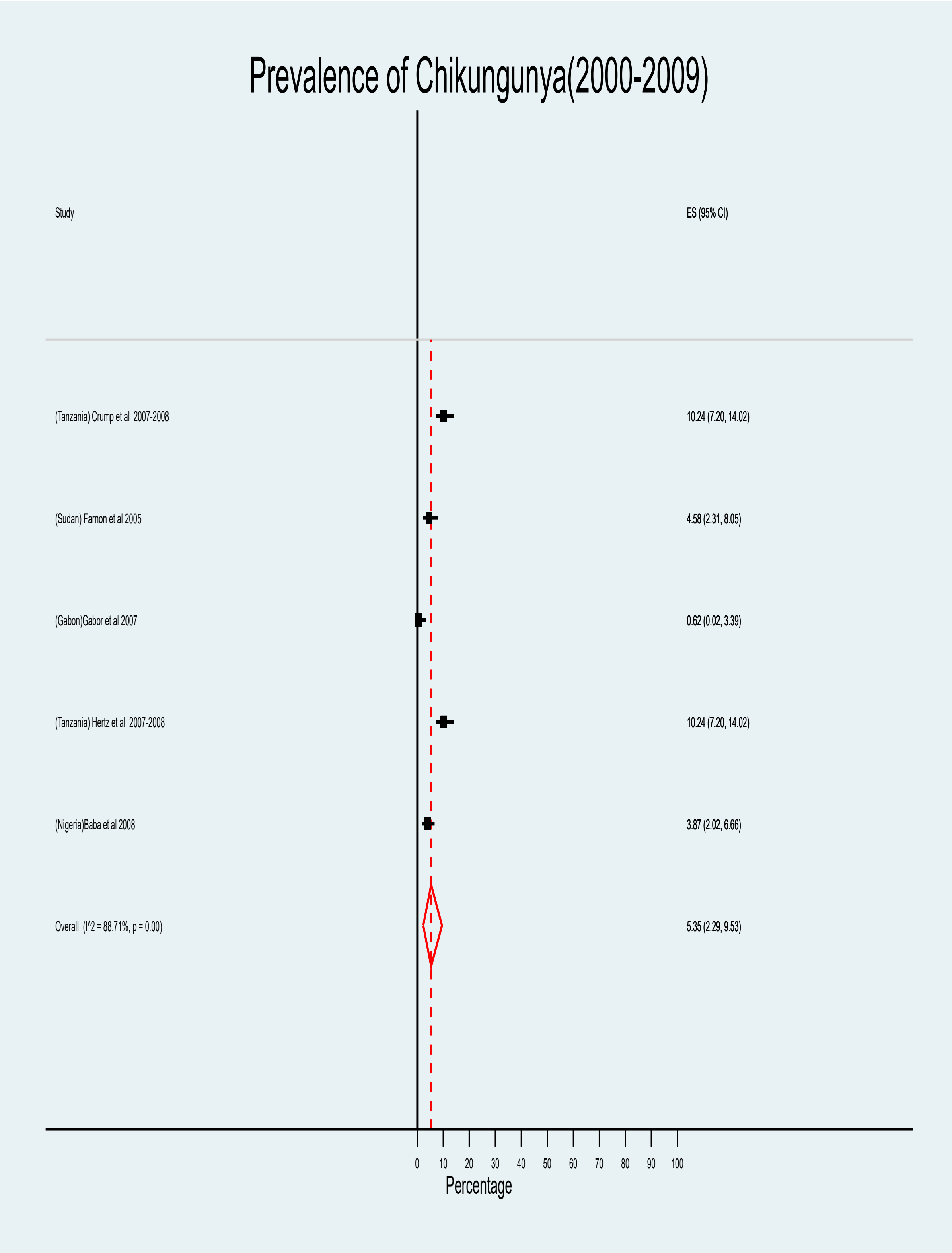

Figure S2a. Forest plot showing the pooled prevalence of chikungunya from 2000 to 2009.

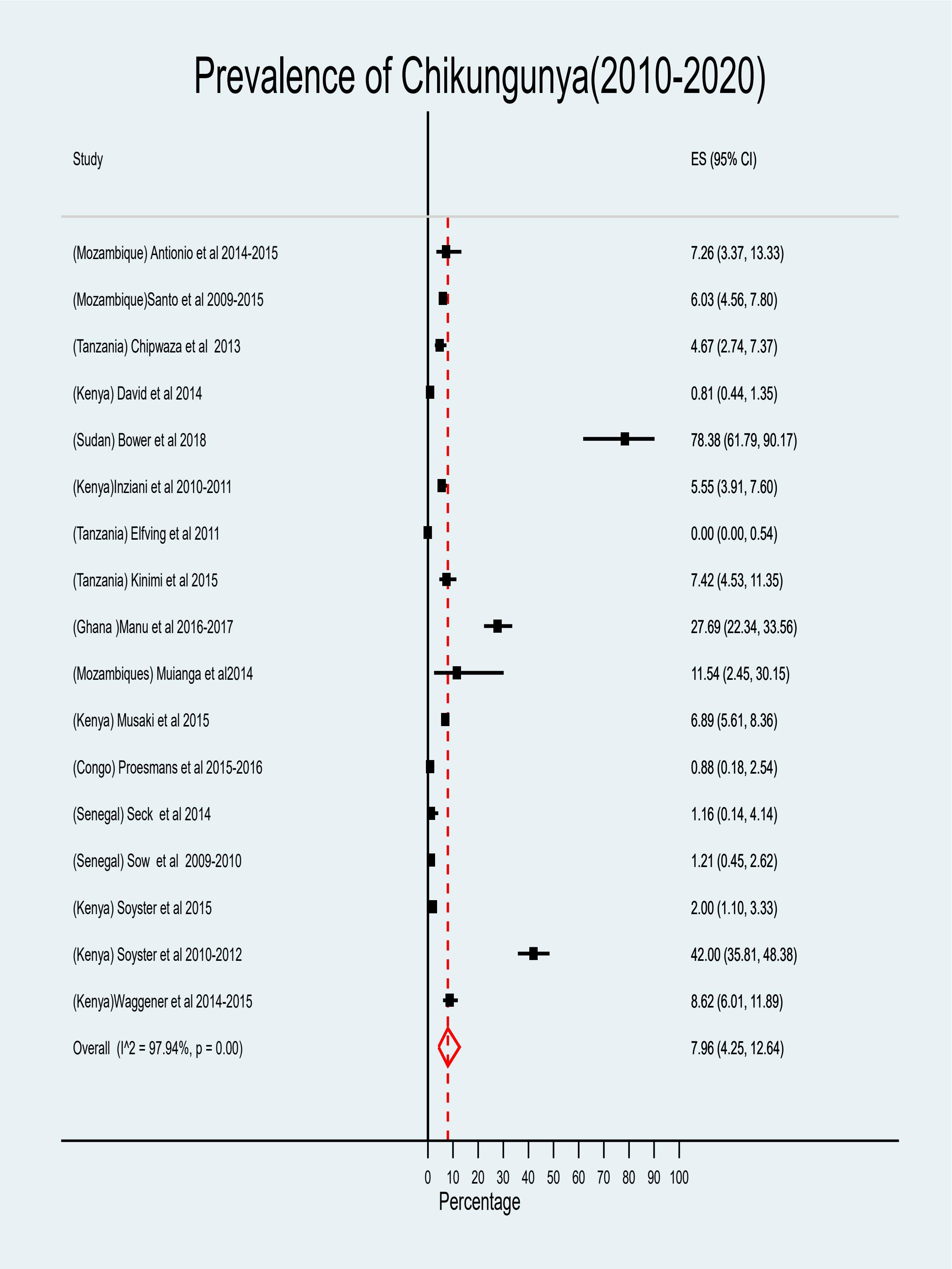

Figure S2b. Forest plot showing the pooled prevalence of chikungunya from 2010 to 2020.
