# Supplementary material for: Seroprevalence of Dengue and Chikungunya Virus Infections in Children Living in Sub-Saharan Africa: Systematic Review and Meta-Analysis
Source: Children (Basel). 2023 Oct 7;10(10):1662. doi: 10.3390/children10101662 (PMC10605353; doi:10.3390/children10101662)
Supplement: Supplementary file 1 [file children-10-01662-s001.zip › Table S1. Arboviruses in Sub-Saharan African children combined data search strategies.pdf]

Table S1. Arboviruses in Sub-Saharan African children combined data search strategies.

| S/no | Search strategiy |
|---|---|
| 1. | **Ovid MEDLINE® ALL**<br>Search Run: June 28, 2019; updated through September 10, 2019 and on February 12, 2022<br><br>1 exp Dengue/<br>2 dengue virus/<br>3 flaviviridae infections/ or flavivirus infections/<br>4 togaviridae infections/ or alphavirus infections/<br>5 Chikungunya Fever/<br>6 Chikungunya virus/<br>7 Arbovirus Infections/<br>8 or/1-19<br>9 exp Africa/<br>10 8 and 9<br>11 limit 10 to "all child (0 to 19 years)"<br>12 ((fever* or infectio* or virus* or viral) adj2 (dengue* or breakbone or "break-bone" or flavivir* or or togaviridae or alphavir* or chikungunya or "chikun-gunya" or arbovir*)).mp.<br>13 (Africa* or Algeria or Angola or Benin or Botswana or "Burkina Faso" or Burundi or Cameroon or "Canary Islands" or "Cape Verde" or "Central African Republic" or Chad or Comoros or Congo or "Democratic Republic of Congo" or Djibouti or Egypt or "Equatorial Guinea" or Eritrea or Ethiopia or Gabon or Gambia or Ghana or Guinea or "Guinea Bissau" or "Ivory Coast" or "Cote d'Ivoire" or Jamahiriya or Jamahiryia or Kenya or Lesotho or Liberia or Libya or Libia or Madagascar or Malawi or Mali or Mauritania or Mauritius or Mayote or Morocco or Mozambique or Mocambique or Namibia or Niger or Nigeria or Principe or Reunion or Rwanda or "Sao Tome" or Senegal or Seychelles or "Sierra Leone" or Somalia or "South Africa" or "St Helena" or Sudan or Swaziland or Tanzania or Togo or Tunisia or Uganda or "Western Sahara" or Zaire or Zambia or Zimbabwe or "Central Africa" or "Central African" or "West Africa" or "West African" or "Western Africa" or "Western African" or "East Africa" or "East African" or "Eastern Africa" or "Eastern African" or "North Africa" or "North African" or "Northern Africa" or "Northern African" or "South African" or "Southern Africa" or "Southern African" or "sub Saharan Africa" or "sub Saharan African" or "subSaharan Africa" or "subSaharan African").mp.<br>14 12 and 13<br>15 (child* or stepchild* or step-child* or kid or kids or girl or girls or boy or boys or teen* or youth* or youngster* or adolescent* or adolescence or preschool* or pre-school* or kindergarten* or school* or juvenile* or minors or p?ediatric* or PICU).mp.<br>16 14 and 15<br>17 11 or 16 |

| | |
|---|---|
| | 18 (vaccin* or immuniz* or immunis*).ti.<br>19 exp *Vaccines/<br>20 exp *Immunization/<br>21 or/18-20<br>22 17 not 21<br>23 limit 22 to english language<br>24 limit 33 to yr="2009 -Current" |
| 2. | **Ovid Embase**<br>Search Run: June 28, 2019; updated through September 10, 2019 and on February 12, 2022<br><br>1 exp dengue/<br>2 exp Dengue virus/<br>3 Flaviviridae infection/<br>4 Flavivirus infection/<br>5 togavirus infection/<br>6 Alphavirus infection/<br>7 chikungunya/<br>8 chikungunya virus/<br>9 or/1-8<br>10 exp Africa/<br>11 9 and 10<br>12 limit 11 to (infant or child or preschool child <1 to 6 years> or school child <7 to 12 years> or adolescent <13 to 19 years>)<br>13 ((fever* or infectio* or virus* or viral) adj2 (dengue* or breakbone or "break-bone" or flavivir* or togaviridae or alphavir* or chikungunya or "chikun-gunya"  or arbovir*)).mp.<br>14 (Africa* or Algeria or Angola or Benin or Botswana or "Burkina Faso" or Burundi or Cameroon or "Canary Islands" or "Cape Verde" or "Central African Republic" or Chad or Comoros or Congo or "Democratic Republic of Congo" or Djibouti or Egypt or "Equatorial Guinea" or Eritrea or Ethiopia or Gabon or Gambia or Ghana or Guinea or "Guinea Bissau" or "Ivory Coast" or "Cote d’Ivoire" or Jamahiriya or Jamahiryia or Kenya or Lesotho or Liberia or Libya or Libia or Madagascar or Malawi or Mali or Mauritania or Mauritius or Mayote or Morocco or Mozambique or Mocambique or Namibia or Niger or Nigeria or Principe or Reunion or Rwanda or "Sao Tome" or Senegal or Seychelles or "Sierra Leone" or Somalia or "South Africa" or "St Helena" or Sudan or Swaziland or Tanzania or Togo or Tunisia or Uganda or "Western Sahara" or Zaire or Zambia or Zimbabwe or "Central Africa" or "Central African" or "West Africa" or "West African" or "Western Africa" or "Western African" or "East Africa" or "East African" or "Eastern Africa" or "Eastern African" or "North Africa" or "North African" or "Northern Africa" or "Northern African" or "South African" or "Southern Africa" or "Southern African" or "sub |

| | |
|---|---|
| | Saharan Africa" or "sub Saharan African" or "subSaharan Africa" or "subSaharan African").mp.<br>15 13 and 14<br>16 (child* or stepchild* or step-child* or kid or kids or girl or girls or boy or boys or teen* or youth* or youngster* or adolescent* or adolescence or preschool* or pre-school* or kindergarten* or school* or juvenile* or minors or p?ediatric* or PICU).mp.<br>17 15 and 16<br>18 12 or 17<br>19 (vaccin* or immuniz* or immunis*).ti.<br>20 exp *vaccine/<br>21 exp *immunization/<br>22 or/19-21<br>23 18 not 22<br>24 limit 35 to english language<br>25 limit 36 to yr="2009 -Current" |
| 3. | **Cochrane Library (Wiley)**<br>Search Run: June 28, 2019; updated through September 10, 2019 and on February 12, 2022<br><br>ID Search Hits<br>#1 MeSH descriptor: [Dengue] explode all trees<br>#2 MeSH descriptor: [Dengue Virus] this term only<br>#3 MeSH descriptor: [Flaviviridae Infections] this term only<br>#4 MeSH descriptor: [Flavivirus Infections] this term only<br>#5 MeSH descriptor: [Togaviridae Infections] this term only<br>#6 MeSH descriptor: [Alphavirus Infections] this term only<br>#7 MeSH descriptor: [Chikungunya Fever] this term only<br>#8 MeSH descriptor: [Chikungunya virus] this term only<br>#9 MeSH descriptor: [Arbovirus Infections] this term only<br>#10 {OR #1-#9}<br>#11 MeSH descriptor: [Africa] explode all trees<br>#12 #10 AND #11<br>#13 MeSH descriptor: [Adolescent] explode all trees<br>#14 MeSH descriptor: [Child] explode all trees<br>#15 MeSH descriptor: [Infant] explode all trees<br>#16 {OR #13-#15}<br>#17 #12 AND #16<br>#18 (fever* or infectio*or virus* or viral) near/2 (dengue* or breakbone or "break-bone" or flavivir* or togaviridae or alphavir* or chikungunya or "chikun-gunya" or arbovir*):ti,ab,kw<br>#19 (Africa* or Algeria or Angola or Benin or Botswana or "Burkina Faso" or Burundi or Cameroon or "Canary Islands" or "Cape Verde" or "Central African Republic" or Chad or Comoros or Congo or "Democratic Republic of Congo" or Djibouti or Egypt or "Equatorial Guinea" or Eritrea or Ethiopia or Gabon or Gambia or Ghana or Guinea or "Guinea Bissau" or "Ivory Coast" or "Cote d'Ivoire" or |

| | |
|---|---|
| | Jamahiriya or Jamahiryia or Kenya or Lesotho or Liberia or Libya or Libia or Madagascar or Malawi or Mali or Mauritania or Mauritius or Mayote or Morocco or Mozambique or Mocambique or Namibia or Niger or Nigeria or Principe or Reunion or Rwanda or "Sao Tome" or Senegal or Seychelles or "Sierra Leone" or Somalia or "South Africa" or "St Helena" or Sudan or Swaziland or Tanzania or Togo or Tunisia or Uganda or "Western Sahara" or Zaire or Zambia or Zimbabwe or "Central Africa" or "Central African" or "West Africa" or "West African" or "Western Africa" or "Western African" or "East Africa" or "East African" or "Eastern Africa" or "Eastern African" or "North Africa" or "North African" or "Northern Africa" or "Northern African" or "South African" or "Southern Africa" or "Southern African" or "sub Saharan Africa" or "sub Saharan African" or "subSaharan Africa" or "subSaharan African"):ti,ab,kw<br>#20 (child* or stepchild* or step-child* or kid or kids or girl or girls or boy or boys or teen* or youth* or youngster* or adolescent* or adolescence or preschool* or pre-school* or kindergarten* or school* or juvenile* or minors or p*diatric* or PICU):ti,ab,kw<br>#21 {AND #18-#20}<br>#22 #17 OR #21<br>#23 (vaccin* or immuniz* or immunis*):ti<br>#24 #22 NOT #23 with Cochrane Library publication date Between Jan 2009 and February 12, 2022. |
