# Supplementary material for: Seroprevalence of Dengue and Chikungunya Virus Infections in Children Living in Sub-Saharan Africa: Systematic Review and Meta-Analysis
Source: Children (Basel). 2023 Oct 7;10(10):1662. doi: 10.3390/children10101662 (PMC10605353; doi:10.3390/children10101662)
Supplement: Supplementary file 1 [file children-10-01662-s001.zip › Table S2.Quality assessment checklist.pdf]

**Table S2.** Quality assessment checklist.[a]

| First Author | Publication Year | Reference Number | Q1 | Q2 | Q3 | Q4 | Q5 | Q6 | Q7 | Q8 | Total "0s" for Q1-Q9[b] |
|---|---|---|---|---|---|---|---|---|---|---|---|
| **M. Baba** | 2013 | 63 | 0 | 1 | 0 | 0 | 0 | 0 | 0 | 0 | 7 |
| **J. Waggener** | 2017 | 33 | 0 | 1 | 0 | 0 | 0 | 0 | 0 | 0 | 7 |
| **A.C. Willcox** | 2018 | 43 | 0 | 0 | 0 | 0 | 0 | 0 | 0 | 0 | 8 |
| **S. Proesmans** | 2019 | 50 | 0 | 0 | 0 | 0 | 0 | 1 | 0 | 0 | 7 |
| **J.T. Hertz et al** | 2012 | 35 | 0 | 1 | 0 | 0 | 1 | 1 | 0 | 0 | 5 |
| **E.N. Grossi-Soyster** | 2015 | 7 | 0 | 0 | 0 | 0 | 0 | 0 | 0 | 3 | 7 |
| **F. Vairo** | 2016 | 30 | 0 | 1 | 0 | 0 | 0 | 0 | 0 | 0 | 7 |
| **V.S. Antonio** | 2018 | 38 | 0 | 0 | 0 | 0 | 1 | 0 | 0 | 0 | 7 |
| **A Muianga** | 2017 | 22 | 0 | 0 | 0 | 0 | 0 | 0 | 0 | 0 | 8 |
| **S.K. Manu** | 2019 | 22 | 0 | 1 | 0 | 0 | 0 | 1 | 0 | 0 | 6 |
| **B Chipwaza** | 2014 | 23 | 0 | 1 | 0 | 0 | 0 | 0 | 0 | 0 | 7 |
| **E.N. Grossi-Soyster** | 2017 | 29 | 0 | 0 | 0 | 0 | 0 | 0 | 0 | 0 | 8 |
| **A.B. Onoja** | 2016 | 55 | 0 | 1 | 0 | 0 | 0 | 0 | 0 | 0 | 7 |
| **V.S. Antonio** | 2019 | 39 | 0 | 0 | 0 | 0 | 0 | 1 | 0 | 0 | 7 |
| **S.B. Tchuandom** | 2019 | 48 | 0 | 0 | 0 | 0 | 0 | 1 | 0 | 0 | 7 |
| **D.M. Vu** | 2016 | 26 | 0 | 0 | 0 | 0 | 0 | 0 | 0 | 0 | 8 |
| **A Malik** | 2010 | 51 | 0 | 1 | 0 | 0 | 0 | 1 | 0 | 0 | 6 |
| **J.M. Blaylock** | 2011 | 32 | 0 | 0 | 0 | 0 | 1 | 1 | 0 | 0 | 6 |
| **J.A. Crump** | 2013 | 34 | 0 | 1 | 0 | 0 | 0 | 0 | 0 | 0 | 7 |
| **M. Inziani** | 2019 | 36 | 0 | 0 | 0 | 0 | 0 | 0 | 0 | 0 | 8 |
| **S.B. Tchuandom** | 2018 | 49 | 1 | 0 | 1 | 0 | 0 | 1 | 0 | 0 | 5 |
| **D.M. Vu** | 2017 | 41 | 0 | 0 | 0 | 0 | 0 | 0 | 0 | 0 | 8 |
| **D.M. Vu** | 2016 | 42 | 0 | 0 | 0 | 0 | 0 | 0 | 0 | 0 | 8 |

| | | | | | | | | | | | |
|---|---|---|---|---|---|---|---|---|---|---|---|
| **K. Elfving** | 2016 | 20 | 0 | 0 | 0 | 0 | 0 | 0 | 0 | 0 | 8 |
| **M. Demanou** | 2014 | 47 | 0 | 0 | 0 | 0 | 0 | 0 | 0 | 0 | 8 |
| **B.A. Ndenga** | 2015 | 24 | 0 | 0 | 0 | 0 | 1 | 1 | 0 | 0 | 6 |
| **S.K. Musak** | 2018 | 37 | 0 | 0 | 0 | 2 | 0 | 0 | 0 | 0 | 7 |
| **A. Sow** | 2014 | 56 | 0 | 0 | 0 | 0 | 0 | 0 | 0 | 0 | 8 |
| **N. Camara** | 2018 | 25 | 0 | 0 | 0 | 0 | 0 | 1 | 1 | 0 | 6 |
| **E.C. Farnon** | 2010 | 53 | 1 | 1 | 0 | 0 | 0 | 0 | 1 | 0 | 5 |
| **S.A. Nassar** | 2019 | 54 | 0 | 1 | 0 | 0 | 0 | 0 | 0 | 0 | 7 |
| **H.G. Boris** | 2021 | 58 | 0 | 0 | 0 | 0 | 1 | 0 | 0 | 1 | 6 |
| **I. Dieng** | 2021 | 59 | 0 | 0 | 0 | 0 | 0 | 0 | 0 | 0 | 8 |
| **J.K. Lim** | 2019 | 45 | 0 | 0 | 1 | 0 | 0 | 0 | 0 | 1 | 6 |
| **J.K. Lim** | 2020 | 31 | 0 | 0 | 0 | 1 | 0 | 0 | 0 | 0 | 7 |
| **J.K. Lim** | 2021 | 60 | 0 | 0 | 0 | 0 | 0 | 1 | 0 | 0 | 7 |
| **J.K. Lim** | 2021 | 61 | 0 | 0 | 0 | 0 | 1 | 0 | 0 | 0 | 7 |
| **H.S. TchetgnaI** | 2021 | 44 | 0 | 0 | 0 | 0 | 0 | 1 | 0 | 0 | 7 |
| **E. Kinimi** | 2018 | 27 | 0 | 0 | 0 | 1 | 1 | 0 | 0 | 0 | 6 |
| **O.M. Kolawole** | 2017 | 65 | 0 | 0 | 0 | 1 | 0 | 0 | 0 | 0 | 7 |
| **J.J. Gabor** | 2016 | 46 | 0 | 0 | 0 | 0 | 1 | 0 | 0 | 0 | 7 |
| **M.C. Seck** | 2019 | 62 | 0 | 0 | 0 | 0 | 1 | 1 | 0 | 0 | 6 |
| **F. Adedayo** | 2013 | 21 | 0 | 0 | 0 | 0 | 0 | 0 | 1 | 0 | 7 |
| **A. Adam** | 2016 | 52 | 0 | 0 | 1 | 0 | 0 | 0 | 0 | 0 | 7 |
| **H. Bower** | 2021 | 58 | 0 | 0 | 0 | 1 | 0 | 0 | 0 | 0 | 8 |
| **O.A. Adesina** | 2016 | 57 | 0 | 0 | 0 | 0 | 0 | 0 | 0 | 0 | 8 |
| **D.M. Vu** | 2017 | 42 | 0 | 0 | 0 | 0 | 1 | 0 | 0 | 0 | 7 |

[a]The quality of each study was evaluated using the Joanna Briggs Institute Critical Appraisal checklist, which uses a 9-item list. Item were scored: “0” if answered “no” or “unclear” and “1” if answered “yes.”

[b]Quality assessment was defined as: high risk, 0-3; moderate risk, 4-6; and low risk, 7-9.
