# Supplementary material for: Seroprevalence of Dengue and Chikungunya Virus Infections in Children Living in Sub-Saharan Africa: Systematic Review and Meta-Analysis
Source: Children (Basel). 2023 Oct 7;10(10):1662. doi: 10.3390/children10101662 (PMC10605353; doi:10.3390/children10101662)
Supplement: Supplementary file 1 [file children-10-01662-s001.zip › Table S3. Seroprevalence of dengue and chikungunya by study area (urban vs rural) and by study site (hospital vs community.pdf]

Table S3. Seroprevalence of dengue and chikungunya by study area (urban vs rural) and by study site (hospital vs community).

Variable	Dengue (%) 95%CI	Chikungunya (%)
**Study Area**		
**Urban**	19.3 (11.2,28.9)	11.2 (5.1,19.2)
**Rural**	12.1 (5.27,21.0)	4.6 (2.8,6.8)
**Both**	1.2 (0.6,1.9)	0
**Study site**		
**Hospital**	18.4 (11.9,25.8)	8.2 (4.6,12.7)
**Community**	6.1 (0.0,25,9)	8.1 (1.5.19.2)
**Both**	6.1 (4.6,12.7)	0
