# Supplementary material for: Seroprevalence of Dengue and Chikungunya Virus Infections in Children Living in Sub-Saharan Africa: Systematic Review and Meta-Analysis
Source: Children (Basel). 2023 Oct 7;10(10):1662. doi: 10.3390/children10101662 (PMC10605353; doi:10.3390/children10101662)
Supplement: Supplementary file 1 [file children-10-01662-s001.zip › Table S4. Laboratory methods used to diagnose arbovirus infections..pdf]

**Table S4.** Laboratory methods used to diagnose arbovirus infections.

| First Author | Year Conducted | Region | Country | DENV | CHKV | Ref |
|---|---|---|---|---|---|---|
| **K. Elfving** | 2011 | Eastern | Tanzania | PCR | PCR | [20] |
| **F. Adedayo** | NA | Western | Nigeria | IgM-ELISA | NA | [21] |
| **A. Muianga** | 2014 | Eastern | Mozambique | NA | IgM-ELISA, IgG-ELISA, RT-PCR | [22] |
| **B. Chipwaza** | 2013 | Eastern | Tanzania | IgM-ELISA, IgG-ELISA, RT-PCR | IgM-ELISA, IgG-ELISA, RT-PCR | [23] |
| **B.A. Ndenga** | NA | Eastern | Kenya | PCR | NA | [24] |
| **N. Camara** | 2014 | Eastern | Tanzania | NSI-RDT, IgM-ELISA | NA | [25] |
| **D.M. Vu** | 2009-2011 | Eastern | Kenya | NU | NA | [26] |
| **E. Kinimi** | 2015 | Eastern | Tanzania | NA | IgM-ELISA, IgG-ELISA | [27] |
| **E.N. Grossi-Soyster** | 2010- 2012 | Eastern | Kenya | IgG-ELISA, PRNT | PRNT | [28] |
| **E.N. Grossi-Soyster** | 2015 | Eastern | Kenya | IgG-ELISA | NA | [29] |
| **F. Vairo** | 2014 | Eastern | Tanzania | RT-PCR | NA | [30] |
| **J.K. Lim** | 2016-2017 | Eastern | Kenya | IgM–ELISA, IgG-ELISA, RT-PCR | NA | [31] |
| **J.M. Blaylock** | NA | Eastern | Kenya | IgG-ELISA | NA | [32] |
| **J. Waggener** | 2014-2015 | Eastern | Kenya | PCR | RT-PCR | [33] |
| **J.A. Crump** | 2007-2008 | Eastern | Tanzania | PCR | PCR | [34] |
| **J.T. Hertz** | 2007-2008 | Eastern | Tanzania | RT-PCR | IgM-ELISA, IgG-ELISA, RT-PCR | [35] |
| **M. Inziani** | 2010-2011 | Eastern | Kenya | IgM-ELISA, IgG-ELISA | IgM-ELISA, IgG-ELISA | [36] |
| **S.K. Musak** | 2015 | Eastern | Kenya | IgG-ELISA | IgG-ELISA | [37] |
| **V.S. Antonio** | 2015-2016 | Eastern | Mozambique | NA | IgM-ELISA, IgG-ELISA | [38] |
| **V.S. Antonio** | 2009– 2015 | Eastern | Mozambique | NA | IgM-ELISA, IgG-ELISA | [39] |
| **D.M. Vu** | 2014-2015 | Eastern | Kenya | RT-PCR | NA | [40] |
| **D.M. Vu** | NA | Eastern | Kenya | RT-PCR | NA | [41] |
| **D.M. Vu** | 2014-2018 | Eastern | Kenya | RT-PCR | RT-PCR | [42] |
| **A.C. Willcox** | 2013-2014 | Central | Congo | IgG-ELISA | NA | [43] |
| **H.S. TchetgnaI** | 2020 | Central | Cameroon | RT-PCR | NA | [44] |

| | | | | | | |
|---|---|---|---|---|---|---|
| **J.K. Lim** | 2015-2016 | Central | Gabon | IgM–ELISA, IgG-ELISA, RT-PCR | NA | [45] |
| **J.J. Gabor** | 2007 | Central | Gabon | IgG-ELISA | IgG-ELISA | [46] |
| **M. Demanou** | 2006-2007 | Central | Cameroon | IgM–ELISA, IgG-ELISA, PRNT | NA | [47] |
| **S.B. Tchuandom** | 2016- 2017 | Central | Cameroon | IgM-ELISA | NA | [48] |
| **S.B. Tchuandom** | 2016-2017 | Central | Cameroon | NSI-RDT, IgM-ELISA, IgG-ELISA | IgM-ELISA | [49] |
| **S. Proesmans** | 2015-2016 | Central | Congo | RT-PCR | NSI-RDT, IFA, IgM-ELISA, IgG-ELISA, RT-PCR | [50] |
| **A. Malik** | 2004-2005 | Northern | Sudan | IgM-ELISA, RT-PCR | NA | [51] |
| **A. Adam** | 2012-2013 | Northern | Sudan | IgM–ELISA, IgG-ELISA | NA | [52] |
| **E.C. Farnon** | 2005 | Northern | Sudan | NA | IgM-ELISA, IgG-ELISA | [53] |
| **H. Bower** | 2018 | Northern | Sudan | RT-PCR | RT-PCR | [54] |
| **A.B. Onoja** | 2014 | Western | Nigeria | RT-PCR | NA | [55] |
| **A. Sow** | 2009-2010 | Western | Senegal | NA | IgM-ELISA, IgG-ELISA, RT-PCR | [56] |
| **O. A. Adesina** | NA | Western | Nigeria | IgG-ELISA | NA | [57] |
| **H.G. Boris** | 2015-2016 | Western | Senegal | PCR | NA | [58] |
| **I. Dieng** | 2017 | Western | Senegal | RT-PCR | NA | [59] |
| **J.K. Lim** | 2016 | Western | Burkina Faso | IgM–ELISA, IgG-ELISA, RT-PCR | NA | [60] |
| **J.K. Lim** | 2015-2017 | Western | Burkina Faso | IgM-ELISA | NA | [61] |
| **M.C. Seck** | 2014 | Western | Senegal | NA | IgM-ELISA | [62] |
| **M. Baba** | 2008 | Western | Nigeria | PRNT | RT-PCR, PRNT | [63] |
| **S.A. Nassar** | 2015-2016 | Western | Nigeria | IgM–ELISA, IgG-ELISA | NA | [64] |
| **O.M. Kolawole** | 2016 | Western | Nigeria | RT-PCR | NA | [65] |
| **S.K. Manu** | 2016-2017 | Western | Ghana | NA | IgM-ELISA, IgG-ELISA | [66] |

KEY: NA, not available; DENV, dengue virus; CHIKV, chikungunya virus; PCR, polymerase chain reaction; RT-PCR, reverse transcription-polymerase chain reaction; IgG-ELISA and IgM-ELISA, enzyme-linked immunosorbent assays that detect antigen-specific IgG and IgM, respectively; NU, neutralizing antibodies; NSI-RDT, non-structural protein 1 antigen-rapid diagnostic test; IFA, indirect fluorescent antibody; PRNT, plaque-reduction neutralization test.
